# Impacts of Reduced Stocking Density on Broiler Welfare During Transport: Evidence from a Hungarian Study Under Moderate Climatic Conditions

**DOI:** 10.3390/ani15213066

**Published:** 2025-10-22

**Authors:** László Szőllősi, Dániel Fróna, Laura Mihály-Karnai, Attila Csorbai

**Affiliations:** 1 Institute of Economics, Faculty of Economics and Business, University of Debrecen, Böszörményi Str. 138, H-4032 Debrecen, Hungary; frona.daniel@econ.unideb.hu (D.F.); karnai.laura@econ.unideb.hu (L.M.-K.); 2 Hungarian Poultry Product Board, Páva Str. 8, H-1094 Budapest, Hungary; csorbai.attila@magyarbaromfi.hu

**Keywords:** slaughter chicken transport, animal welfare legislation, stocking density, dead-on-arrival (DOA), physical injuries, condemnations

## Abstract

**Simple Summary:**

Transport conditions have a major impact on chicken welfare, meat quality, and farm economics. Recently, the European Food Safety Authority (EFSA) has recommended providing broiler chickens with more space during transport to improve welfare. Our study tested this under commercial conditions on a Hungarian farm, comparing the standard EU stocking density with the reduced density suggested by the EFSA. The trial was conducted in spring under mild temperatures and short transport distances. Surprisingly, chickens transported with more space (reduced density) showed poorer outcomes, including a higher incidence of mortality, injuries, and carcass rejections. These findings suggest that, under moderate European temperatures, giving chickens extra space in transport crates may actually increase the risk of injury and death. Further research under more extreme temperatures, longer distances, and with alternative crate systems is recommended before such recommendations are widely implemented.

**Abstract:**

Broiler transport density plays a crucial role in animal welfare, meat quality, and economic efficiency. The European Food Safety Authority (EFSA) has recently recommended increased space allowances for broilers during transport to improve welfare. This study statistically evaluated the effects of reduced stocking density under commercial Hungarian conditions. A total of 176,198 Ross 308 heavyweight broilers were transported over a 19 km distance at moderate springtime temperature (7–13 °C) using 33 trucks, of which 14 (7 control and 7 test) were included in the comparison. Control trucks followed the EU-regulated density (160 cm^2^/kg; 5610 birds/truck), while test trucks applied the lower EFSA-recommended density (200–210 cm^2^/kg; 4334 birds/truck). Dead-on-arrival (DOA) birds and carcass condemnations were evaluated for all transported birds, while injuries and bruising were assessed by sampling 277 carcasses per truck, consistent with industrial auditing practice. Significant differences were observed between the two treatment groups. The reduced-density group showed higher rates of DOA birds (0.61% vs. 0.36% in the control, a 69% relative increase), more wing injuries (6.91% vs. 4.28%, +61%), more bruising (6.76% vs. 3.40%, +98%), and a higher rate of carcass condemnations (0.78% vs. 0.57%, +38%). These findings indicate that increasing space allowance during transport may not improve welfare under moderate continental conditions and may even increase injury risk, with potential economic and environmental consequences for stakeholders. The results highlight the need for further research covering longer journey durations, wider temperature ranges, and alternative crate designs before the broad implementation of the EFSA-recommended transport densities.

## 1. Introduction

Poultry production is one of the fastest-growing agricultural sectors worldwide and a major source of affordable animal protein [1,2]. Global poultry meat production reached 150 million tonnes in 2024, with chicken dominating (85–90%) [3]. Growth has been driven by efficient production cycles and strong demand, although highly pathogenic avian influenza (HPAI) continues to pose challenges [4,5,6]. Despite this, production is expected to rise further in the future [7]. China, the United States, Brazil, and the European Union (EU) together account for 53.8% of global supply [8]. In the EU, production exceeds 14 million tonnes annually, with chicken representing 80%. Poland, Spain, France, and Germany contribute over half of total output, while Central and Eastern Europe show growth potential [9]. The EU poultry sector benefits from lower greenhouse gas emissions compared with red meat [10,11], declining antibiotic use [12] and high animal welfare standards. However, competitiveness is limited by higher labour, feed, and compliance costs than in Brazil or the US [13,14,15]. At the same time, technological development, consumer trust, and the integrated EU market provide stability [15,16].

Poultry meat remains a strategic product for food security and trade, where transportation plays a critical role in both efficiency and animal welfare. Beyond economic performance, transport conditions directly affect bird welfare and meat quality, requiring continuous adaptation to new welfare and sustainability expectations [17,18].

### 1.1. EU Regulation and Innovation in Poultry Transport

The European Union’s Green Deal aims to enhance food system sustainability, mitigate climate change, and strengthen animal welfare. Within this strategic framework, the Farm to Fork strategy seeks to make the entire food chain—from production to consumption—more sustainable and ethical in an integrated manner [19].

The main goal is to implement evidence-based animal welfare practices, particularly during transport. In 2020, the European Commission launched a comprehensive review of animal welfare legislation, focusing on Regulation (EC) No 1/2005, which defines legal and technical requirements for live animal transport, including poultry [20]. In December 2023, the Commission adopted a new regulatory proposal introducing more precise, science-based requirements on transport conditions, journey duration, and heat stress prevention [20]. The proposal is supported by the European Food Safety Authority (EFSA) scientific opinions [21] and the European Commission’s comprehensive assessment [20,22], considering impacts on animal welfare, economic performance, environmental sustainability, and societal well-being. Technological innovations, such as aerodynamically ventilated, heat-resistant, and sensor-equipped transport crates, have become essential for improving animal comfort, reducing losses, and supporting supply chain sustainability [21,23].

**Figure 1:**
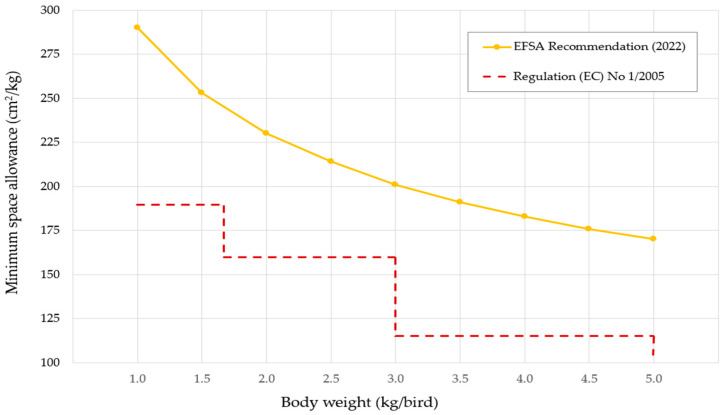
Comparison of minimum space allowances in poultry transport. Source: [21,24].

A key proposed amendment for broiler transport requires that animal space and transport equipment meet at least the minimum standards outlined in Chapter VII of the draft regulation [20]. EU legislation (Section E of Chapter VII of Annex I to Regulation (EC) No 1/2005) defines minimum space allowances per bodyweight category [24]. Depending on the season, the industry generally applies a space allowance of 170 cm^2^/kg for birds weighing 1.6–3.0 kg, slightly above the EU standard of 160 cm^2^/kg, to support animal welfare. Current legislation is widely accepted in practice, providing seasonal flexibility that allows operators to adjust loading densities—for example, maintaining 160 cm^2^/kg under colder conditions or reducing it in warmer periods. EFSA recommendations are significantly higher, ranging from 290 cm^2^/kg for 1 kg birds to 170 cm^2^/kg for 5 kg birds [21], highlighting a gap between current regulations and scientific advice (Figure 1).

The design and materials of compartments (containers and crates) used in poultry transport have evolved considerably in recent decades, driven by stricter animal welfare standards, efforts to reduce transport losses, and the need for greater logistics efficiency. Heavy, metal-framed crates of the past, often featuring sharp edges, have gradually been replaced by modern, ergonomically shaped, lightweight, and durable plastic crates. These innovations not only enhance loading efficiency but also provide better protection for the birds [25,26].

Ventilation and heat-dissipation features are now specifically engineered to minimise thermal stress, particularly in hotspots within the vehicle. Studies have shown that variations in temperature and humidity can lead to significant dead-on-arrival (DOA) rates, especially during the summer months [27]. The aerodynamic design of these modern compartments promotes air circulation and reduces thermal stress, directly supporting both animal welfare and meat quality, while also limiting product losses.

The modular and standardised dimensions of modern crates allow the use of automated loading systems, reducing dependency on manual labour and injuries associated with handling. Their lighter weight contributes to lower fuel consumption, positively impacting the environmental footprint of the transport system. Emerging “smart crate” technology integrates sensors to monitor temperature, humidity, and movement in real time, enabling rapid detection of critical conditions and timely preventive interventions [28]. While still primarily in the experimental phase, these systems are expected to play a pivotal role in future poultry transport, achieving the animal welfare objectives outlined in the Green Deal and the Farm to Fork strategy.

### 1.2. Relationships Between Transport Density, Animal Welfare, and Meat Quality

Different studies have approached poultry transport from multiple perspectives, using a range of parameters to assess both direct and indirect effects on welfare. Some research focuses explicitly on animal welfare indicators, while other studies consider physiological or product-related outcomes that indirectly reflect welfare status.

Poultry transport represents a critical stage in the production chain, influencing animal welfare, economic performance, and meat quality. Birds are exposed to a range of physical and environmental stressors—including extreme temperatures, overcrowding, deprivation of feed and water, vehicle vibrations, noise, instability due to movement, and social stress—that can cause stress, fear, physical injuries, or even mortality. The severity of these effects depends on transport duration, climatic conditions, and handling practices. Among these factors, stocking density is particularly critical, influencing thermal environment, freedom of movement, and physical contact between birds. Optimising stocking density is therefore central to controlling pre-slaughter stress, mortality, and meat quality, which underpin production competitiveness [29,30,31,32].

Previous studies have investigated the effects of different transport densities from welfare, physiological, and meat quality perspectives. Results indicate that very high densities increase thermal stress, mortality, and the incidence of bruising or fractures, whereas excessively low densities can also be unfavourable, especially under cold conditions, by impairing thermal comfort and increasing energy requirements [26,30,32,33,34]. Optimising density is therefore essential to balance welfare protection and economic efficiency.

Heat stress remains the main cause of transport-related mortality—accounting for up to 95% of deaths—while poor ventilation and high density exacerbate these effects [23,35]. At the same time, transport stress has been shown to alter muscle metabolism and biochemical composition, thereby deteriorating meat quality and reducing processing yield [32,36,37,38,39,40,41]. These findings consistently demonstrate that transport density plays a pivotal role not only in safeguarding animal welfare but also in maintaining the sensory and technological quality of poultry meat.

Field studies under various climatic conditions further confirm that the interaction between stocking density, journey duration, and ambient temperature determines overall welfare and product quality outcomes [33,42]. However, evidence from temperate European conditions remains scarce, highlighting the need for region-specific assessments.

### 1.3. Economic Impacts and Production Trade-Offs

Optimising transport density is not only essential for animal welfare and meat quality but also has significant economic implications [43,44]. For producers and integrators, the main objective is to maximise the number of live birds per track to reduce transport costs per kilogram [38]. However, overcrowding increases the risk of mortality, injuries, and stress-induced meat quality deterioration, which leads to direct income losses and inefficiencies in processing [29].

Previous studies [26,38] indicate that while lower transport densities raise logistics costs per animal, they may improve welfare and meat quality and increase consumer acceptance, thus partly compensating for additional expenses. Consequently, excessive transport stress—especially under unfavourable temperature conditions or during long journeys—can cause significant carcass downgrades and profitability losses due to PSE-like meat and drip loss [28,45].

Seasonal effects also play a decisive role. Research comparing different densities under summer and winter conditions found that lower densities improved welfare and reduced injuries in hot weather, whereas higher densities provided better thermal comfort in cold conditions, confirming that the optimal loading rate must balance thermal stress and available space [46].

A recent Hungarian small-scale study [47], conducted in two slaughterhouses using data from two transport trucks per company, assessed the animal welfare, economic, and environmental impacts of reducing broiler transport density to EFSA’s recommended level under winter conditions. Lower density resulted in worse welfare indicators (e.g., higher DOA rates, condemnations, and injuries) in both cases, with no observable welfare benefits. Economically, it increased per-unit transport costs and poultry meat prices, while environmentally, it required more trips, resulting in higher greenhouse gas emissions and water use.

### 1.4. Study Objective

The literature review clearly indicates that broiler transport density plays a crucial role in animal welfare, meat quality, and economic efficiency. Lower transport densities can reduce heat stress, injuries, and DOA rates, while improving meat quality, particularly under adverse environmental conditions. However, excessively low densities may increase energy use and logistics costs, whereas higher densities may provide short-term economic advantages.

The present study aims to statistically evaluate the effects of reduced stocking density during broiler transport, comparing the EU’s current regulatory density with the lower density recommended by the EFSA. The analysis focuses primarily on animal welfare outcomes, including mortality (DOA), limb injuries, bruising, and carcass condemnations, while also providing a simplified assessment of related economic implications.

Data were collected from a Hungarian production site comprising ten houses with a total capacity of approximately 180,000 birds. Building on the findings of a previous small-scale Hungarian study [47] conducted under moderate temperatures, the working hypothesis posits that increasing the available space during broiler transport under moderate environmental conditions is associated with a higher incidence of injuries and a corresponding increase in the proportion of DOA birds.

This large-scale study, involving 33 truckloads, specifically focuses on springtime conditions in Hungary, representing transitional, non-extreme temperatures typical of continental European climates. By investigating moderate climatic conditions, the study aims to provide insights into the effects of transport density under realistic, commercial operating environments, rather than under extreme heat or cold stress scenarios.

## 2. Materials and Methods

### 2.1. Study Design and Procedures

**Figure 2:**
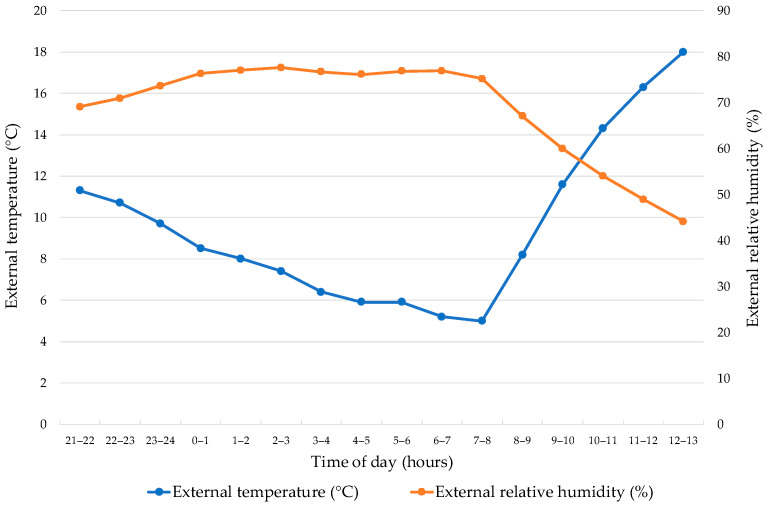
Hourly variation of ambient temperature and relative humidity during broiler transport. Source: Own data (2025).

A total of 176,198 Ross 308 heavyweight broilers (average body weight 3.199 ± 0.035 kg) were transported from a Hungarian farm comprising ten houses to a commercial slaughterhouse located 19 km away. The transport took place between 21:00 on 3 April 2025 and 13:00 on 4 April 2025. During this period, ambient temperature ranged from 5.0 to 18.0 °C (mean 9.34 ± 3.83 °C), and relative humidity varied between 44.1% and 77.6% (mean 69.86 ± 10.82%) (Figure 2).

Catching was performed according to commercial practice using the one-leg technique, and all birds were handled by the same trained catching teams (A, B, C, and D). The Stork Marel Atlas container system (Marel, Garðabær, Island) was used for all transports. Each vehicle (Volvo FH (AB Volvo, Gothenburg, Sweden) + Schwarzmüller RH125 (Schwarzmüller Group, Freinberg, Austria) with CMC Agile loading vehicle (CMC Industries, Cazzago San Martino, Italy)) was equipped with five-tier containers: the lower crates measured 233 × 100 cm (24,000 cm^2^) and the four upper crates 233 × 110 cm (27,000 cm^2^), all with 27 cm crate height and central division. Loading time was approximately 45 min per truck.

A total of 33 trucks were required to transport the entire flock. At the slaughterhouse, consignments were handled separately. Transport data and slaughterhouse results were recorded for all 33 trucks (see Section 2.2 for details). Of these, 7 trucks were loaded at the lower density recommended by the EFSA (Test truck), and 26 trucks at the company’s standard EU-regulated density (Control truck). Due to slaughterhouse scheduling constraints, only the first 14 trucks (7 Control and 7 Test) could be processed within a single shift. Therefore, these 14 trucks, all transported between 21:00 and 03:00 under comparable environmental conditions, were selected for the primary statistical comparison. The remaining 19 trucks were handled and recorded in the same way but processed during the following shift, and their data were excluded from the comparative analysis.

**Figure 3:**
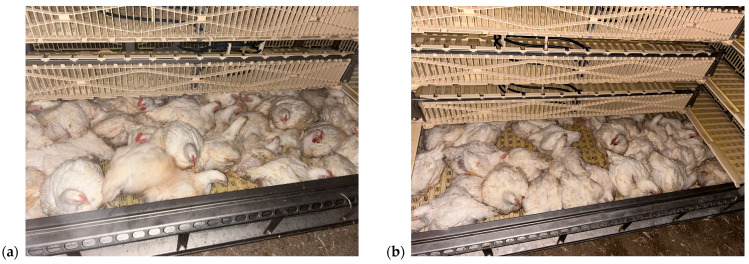
Stork Marel Atlas container loaded according to (**a**) the current EU regulation (lower crate: 47 chickens per crate) and (**b**) the EFSA recommendation (lower crate: 37 chickens per crate). Source: Own data (2025).

Under the current EU regulation (Regulation (EC) No 1/2005) and company practice, Control trucks were loaded at 159–162 cm^2^/kg, corresponding to 47 birds in the lower and 52 birds in the upper crates, for a total of 5610 birds per truck. Test trucks were loaded according to the EFSA’s recommendation at 203–211 cm^2^/kg, corresponding to 37 and 40 birds per lower and upper crate, respectively, for a total of 4334 birds per truck. Consequently, transporting the entire flock at the EFSA-recommended density would have required 41 truckloads instead of 32, i.e., 9 additional trips for the same number of birds. Figure 3 shows the difference in container loading.

To ensure a homogeneous study population and minimize confounding factors, all birds originated from the same farm and were of mixed sex but uniform age and average body weight. The same catching and loading crews (A and B), transport route, and driving style were maintained for all consignments, and all birds were slaughtered and evaluated by the same personnel. Thus, stocking density was the only variable between treatments.

All procedures were carried out in full compliance with EU animal welfare legislation and ethical standards.

### 2.2. Data Collection and Welfare Assessment

For each of the 33 transport trucks, detailed operational and environmental data were recorded, including the total number of birds, total live weight, average body weight, departure and arrival times, transport duration, lairage time at the slaughterhouse, and ambient conditions (external temperature and relative humidity). Categorical variables included catching shift (evening or daytime), catching team (A or B for evening shifts, and C or D for daytime shifts), and slaughterhouse processing shift (1 or 2). These records provided a complete dataset for flock-level analysis.

Animal welfare outcomes were evaluated and expressed as ratios relative to the number of birds transported using four primary indicators:

(1) Mortality during transport, expressed as the number of DOA birds;
(2) Carcass condemnations recorded during post-mortem inspection;
(3) Visible limb (wing or thigh) injuries;
(4) Bruising or hematomas on the carcass surface.

DOA birds and carcass condemnations were assessed for all transported birds in each truck, providing a full-population measure of pre-slaughter welfare and product loss. In contrast, limb injuries and bruising were evaluated through representative sampling, following the auditing standards commonly applied in poultry processing plants.

For each truck, carcass assessment took place approximately midway through the processing of that consignment. Carcasses were continuously observed for one minute by trained quality assurance personnel, corresponding to the throughput of about 277 birds (based on the slaughter line speed of 16,650 birds per hour). The birds were evaluated after stunning and defeathering, ensuring that visible bruises and fractures could be accurately identified. This sampling approach, although partial, reliably reflects the prevalence of visible injuries and bruising under commercial processing conditions and is consistent with standard industrial auditing practice.

Lesions were classified according to standardised definitions:

- Hematomas were defined as vital (ante-mortem) bleedings larger than 20 mm, infiltrating muscle and skin tissue.
- Limb injuries included dislocations, luxations, or fractures, with severe cases recorded when broken bones were visibly protruding.
- Fractures associated with hematomas were also classified as painful injuries.

All visual assessments were performed by experienced quality assurance staff members who routinely participate in internal welfare auditing and are retrained every five years under the supervision of competent veterinary authorities. These procedures ensured consistency and reliability of scoring across all consignments.

### 2.3. Data Analysis

Data from the 33 trucks were analysed using two complementary approaches.

First, a comparative analysis assessed the effects of loading density using data from the first 14 trucks (seven Control and seven Test trucks). Descriptive statistics were produced, followed by a test of normality (Shapiro–Wilk test) and homogeneity of variance (Levene’s test). Depending on distribution and variance, group comparisons used the independent-samples *t*-test, Welch’s *t*-test, or the Mann–Whitney U-test. For significant differences, effect sizes were calculated as Cohen’s d (0.2 = small, 0.5 = medium, 0.8+ = large effect).

Second, to better understand the potential influence of environmental and operational factors under commercial conditions, correlation analyses were performed using data from all 26 Control trucks transported under current EU regulation. This broader dataset allowed the assessment of relationships between transport conditions (temperature, humidity, transport duration, lairage time) and welfare indicators, helping to contextualize variation within routine commercial practice. Pearson correlation was used for normally distributed variables, and Spearman correlation otherwise. Group comparisons across catching and slaughterhouse shifts were conducted using the same statistical tests as above.

All analyses were performed using standard statistical software (IBM SPSS Statistics, version number: 29.0.0.0), with significance set at *p* < 0.05.

## 3. Results

During the experimental period in early April in Hungary, external conditions were typical for the season, with relatively mild temperatures and moderately high humidity levels. Between 21:00 and 03:00, external temperatures ranged from 7.4 to 11.3 °C, while relative humidity varied between 69.1 and 77.6%. Compared with the broader meteorological conditions during the catching and transport of the entire flock, the environmental parameters for the 14 selected trucks used in the comparative analysis showed a narrower range of variation. This ensured that external environmental variation had minimal influence on the comparative outcomes.

Table 1 and Table 2 provide detailed descriptive statistics for the Control and Test transports, including mean, range, median, variance, and standard error values. These data are reported separately to support reproducibility and to assist future researchers in interpreting data distributions, particularly because both parametric and non-parametric tests were used.

Significant differences were observed between the Test and Control loading densities for DOA birds, wing injuries, bruising, and carcass condemnations (Table 3). The effect sizes for these variables were substantial, indicating pronounced differences. Specifically, the proportion of DOA birds was 0.61% in the Test trucks compared to 0.36% in the Control trucks (a 69% relative increase). The proportion of birds with wing injuries was 6.91% vs. 4.28% (+61%), bruising 6.76% vs. 3.40% (+98%), and carcass condemnations 0.78% vs. 0.57% (+38%). Under the examined moderate spring conditions, transport at the reduced stocking density (200 cm^2^/kg) recommended by the EFSA resulted in markedly poorer welfare outcomes than transport under the current EU regulation (160 cm^2^/kg).

For thigh injuries, a medium effect size was observed, though the difference was not statistically significant. No statistically significant differences were found in transport conditions (temperature, humidity, travel duration, or lairage time), which was a prerequisite of the study design. Similarly, no significant differences were observed between the catching teams (A and B) for any of the welfare indicators, confirming the consistency of handling procedures.

Correlation analyses were conducted using data from all 26 Control trucks loaded in compliance with the current EU regulation on stocking density. Table 4 presents descriptive statistics for these 26 broiler transports. In this larger sample, the study period spanned a longer time interval (from 21:00 on 3 April to 13:00 on the following day), with a wider range of external temperatures (5.0–18.0 °C) and relative humidity (44.1–77.6%). Of the 26 transports, 10 involved evening catching shifts (four by Team A and six by Team B), while 16 were carried out during daytime catching shifts (seven by Team C and nine by Team D). With regard to slaughterhouse operations, 12 transports were processed during the first shift and 14 during the second.

This extended dataset allowed us to examine potential associations between environmental and operational factors (temperature, humidity, duration, lairage time, catching team, and slaughter shift) and welfare indicators. Although ambient temperature variation was relatively small, this analysis served to verify that other operational variables did not introduce confounding effects. Such correlations are valuable for contextualizing results under commercial conditions and for informing future large-scale animal transport studies.

Table 5 presents the results of the correlation analysis between transport conditions and animal welfare indicators. Despite the fact that within the sample of 26 transport events, ambient temperature and humidity exhibited considerable variation, no statistically significant associations could be established between transport conditions and animal welfare indicators. The only exception was the relationship between humidity and thigh injuries, where a moderately positive correlation was observed.

Furthermore, no statistically significant differences were detected across different loading shifts or loading teams for any of the welfare parameters examined. Similarly, no statistically significant differences were observed between slaughterhouse shifts with respect to any of the assessed indicators. These findings collectively confirm that the experimental design successfully minimised potential confounding effects, thereby strengthening the validity and interpretability of the comparative analyses.

A simple operational estimate indicates that, for the study flock (176,198 birds), compliance with the EFSA-recommended density (4334 birds per truck) would necessitate 41 truckloads compared to 32 truckloads under the current company practice (5610 birds per truck), i.e., 9 additional trips. Considering the known one-way distance of 19 km, this corresponds to an additional 342 km for the entire consignment. Assuming an average transport cost of 2.1 EUR per kilometre for the company (which is not generalisable, as it depends on vehicle type, wage levels, and other operational factors), this implies an increase of 718 EUR in transport expenditure.

In addition to direct transport costs, it is important to account for the opportunity cost associated with welfare-related losses. Higher transport densities may increase the incidence of DOA birds and carcass condemnations, thereby reducing the marketable output and generating lost revenue. Assuming a processing price of 1.15 EUR per kg live weight, the observed increase of 0.25% in DOA birds and 0.22% in condemnations corresponds to a total estimated loss of 3050 EUR for the study flock.

Consequently, the overall economic impact of adopting the EFSA-recommended density arises from the combined effect of higher transport costs and losses due to mortality and condemnations. These calculations are intended to be illustrative and conservative: a comprehensive economic assessment would require a detailed breakdown of costs (fuel, driver wages, vehicle depreciation, cleaning, lairage, and potential effects on throughput).

## 4. Discussion

This large-scale study was conducted in Hungary under moderate temperatures, involving an entire farm population of almost 180,000 heavyweight broilers transported over a short distance (19 km). The aim was to statistically evaluate the effects of reduced stocking density during broiler transport by comparing transport at the EU’s current regulatory density (160 cm^2^/kg) with transport at the lower density recommended by the EFSA (200 cm^2^/kg). The study focused on direct welfare-related indicators associated with transportation, including DOA birds, condemned carcasses, birds with limb (wing and thigh) injuries, and birds exhibiting bruises.

Significant differences were observed between the two treatment groups. In the reduced-density group, the proportion of DOA birds was 0.61% compared with 0.36% in the control group (a 69% relative increase). Similarly, the proportion of birds with wing injuries was 6.91% versus 4.28% (+61%), bruising was 6.76% versus 3.40% (+98%), and carcass condemnations were 0.78% versus 0.57% (+38%).

The higher incidence of injuries and mortality in the reduced-density group can be explained by increased bird mobility within the crates. Greater available space may have allowed more wing flapping, shifting, and collisions during vehicle movements, leading to mechanical injuries. These findings suggest that, in such conditions, increasing space per bird does not necessarily enhance welfare and may in fact increase the risk of injury.

In addition to this comparative analysis, no significant associations were found between transport conditions, loading, or slaughterhouse shifts and welfare indicators, thereby supporting the study’s methodological aim of minimising confounding factors and strengthening the validity of the results.

In the Hungarian case study [47], which examined heavyweight broiler transport in two companies on a relatively small sample, similar patterns were observed. In the first company, where traditional plastic crates were used, transport at the reduced stocking density recommended by the EFSA resulted in 96% higher DOA rates, 46% higher rates of limb injuries, 45% higher rates of bruising, and 55% higher rates of condemnations compared with transport conducted at the current EU regulatory density. In the second company, where the same Stork Marel Atlas containers were applied as in the present study, the corresponding increases were even more pronounced: 159% higher DOA rates, 57% more limb injuries, 87% more bruising, and 237% more condemnations. Compared with the present findings, the less favourable outcomes of the earlier study were most likely influenced by the considerably longer transport distances (72 and 150 km, respectively) and the lower ambient temperatures prevailing in January. Overall, the results of the present study statistically corroborate and reinforce the findings of the earlier Hungarian case study [47].

Another previous Hungarian survey [48] reported inconclusive outcomes regarding the effects of increased space allowance per bird. In that study, transports with a stocking density below 207 cm^2^/kg showed a DOA rate of 0.6%, while densities above this threshold were associated with a slightly lower rate of 0.49%. However, the study noted that transport-related injuries were more frequent at higher space allowances (0.31% for <207 cm^2^/kg vs. 0.51% for >207 cm^2^/kg), presumably due to collisions and instability during vehicle braking and turning. With greater space, birds have more opportunity for wing flapping and shifting, which in turn increases the risk of liver ruptures, wing and leg fractures, and bruising. Comparable findings were reported in a Portuguese slaughterhouse survey [49], which investigated 64 short-distance transports of broilers with an average body weight of 1.85 kg. The study identified a positive correlation between space allowance and the incidence of bruising: the probability of bruising exceeding 4% increased linearly, from around 20% at 180 cm^2^/kg to 60% at 230 cm^2^/kg. On this basis, the study suggested that transport containers providing less space per bird might reduce bruising, as closer body contact helps stabilise the animals, lowers the risk of falls, and diminishes the need for wing and leg movements to maintain balance.

The results of our study clearly indicate that the EFSA recommendation for increased space per bird during transport does not improve the key animal welfare indicators under moderate temperatures. The Poultry Veterinary Study Group Europe [50] cites recent studies in the United Kingdom [51] and Germany [52], which found that stocking density had no significant effect on DOA rates, one of the most important welfare metrics. Our findings support the PVSGE’s position that reducing stocking density under moderate environmental conditions does not enhance key welfare outcomes. On the contrary, increasing space per bird may exacerbate injuries caused by slipping, wing flapping, and trampling, which not only compromise welfare but also raise the proportion of condemned and injured carcasses at the processing plant, ultimately reducing product yield.

Several studies have reported that increasing stocking density during broiler transport can have detrimental effects on key welfare and product quality indicators, particularly under hot summer conditions. Xing et al. [37] demonstrated that high transport density under heat stress critically compromises meat quality and protein stability. Similarly, Hussnain et al. [39,40] reported that lower transport density (approximately 210 cm^2^/kg) helped mitigate the adverse effects of heat stress by reducing the risk of injuries, metabolic dysfunctions, and deterioration of meat quality, while also exerting a positive influence on the sensory and technological properties of the meat. These findings indicate that the interaction between transport density and environmental temperature plays a decisive role in determining both welfare outcomes and product integrity, highlighting that under heat stress, reduced stocking density may offer protective effects.

While these studies highlight the potential benefits of reduced stocking density under hot summer conditions, our results indicate a contrasting pattern under moderate springtime temperatures (7–13 °C). Specifically, when transport was conducted at the lower densities recommended by the EFSA, higher rates of DOA birds, limb injuries, bruising, and carcass condemnations were observed. This suggests that mandating increased space allowances across all transport scenarios may not universally improve welfare outcomes and could even prove counterproductive under certain environmental conditions. Evidence from previous studies conducted in Canada [53], Republic of Korea [46], Pakistan [42], and Belgian [33] supports this view, showing that higher stocking densities can be advantageous in colder conditions. Our results align with the concept highlighted by Yu et al. [46] that optimal transport density is season-dependent, requiring a careful balance between thermal stress and space availability to preserve both meat quality and animal welfare. Consequently, blanket regulations enforcing lower densities, without considering environmental and economic context, may inadvertently compromise welfare outcomes in continental European temperatures, as demonstrated by the present study.

Taken together, these findings underscore the importance of tailoring regulatory frameworks to climatic and production contexts, rather than applying uniform stocking density requirements across all conditions. Improving animal welfare in poultry transport is therefore not only a scientific and ethical imperative, but also a structural element of the EU’s broader sustainability strategies. The Green Deal and the Farm to Fork strategy both emphasise that welfare improvements must be addressed as part of an integrated food chain approach, linking producers, processors, and consumers. In this context, the 2023 legislative proposal represents an important milestone, providing an opportunity to align animal welfare requirements with technological developments across the poultry sector.

## 5. Conclusions and Recommendations

In recent years, the modernization of poultry transport—particularly the innovative development of crates and transport systems—has closely aligned with the principles of the European Green Deal and the Farm to Fork strategy. These technological innovations aim to enhance animal welfare and production efficiency while supporting environmental sustainability and consumer trust. Integrating these aspects remains essential to maintain the competitiveness and social acceptance of the poultry sector.

This large-scale study, involving 33 truckloads and approximately 180,000 heavy-weight broilers, compared the current EU-regulated stocking density (160 cm^2^/kg; 5610 birds per truck) with the reduced density recommended by the EFSA (200 cm^2^/kg; 4334 birds per truck) under moderate spring conditions (7–13 °C) and short transport distance (19 km). The results clearly showed that reduced stocking density did not improve animal welfare. On the contrary, it was associated with higher rates of DOA birds, wing injuries, bruising, and carcass condemnations. These findings support the hypothesis that increasing the available space during broiler transport under moderate environmental conditions is associated with a higher incidence of injuries and a corresponding increase in the proportion of DOA birds.

From an economic standpoint, adopting the EFSA-recommended density would require additional truckloads, resulting in higher fuel use, labour, and operational costs, alongside a larger environmental footprint. At the same time, the higher mortality and condemnation rates observed under reduced density translate into measurable revenue losses, further weakening economic sustainability.

The study highlights that the European Commission’s proposed draft regulation could negatively affect stakeholders from both economic and environmental perspectives, without clear evidence of welfare benefits. The findings contribute to the practical evaluation of EFSA recommendations and are highly relevant for Hungarian and broader European regulatory contexts. It is recommended that these results be taken into account by the European Commission and policymakers involved in the preparation of animal welfare legislation.

The results of this study should be interpreted within the context of springtime conditions, with ambient temperatures ranging between 7–13 °C, a relatively short transport distance of 19 km (20–25 min), and heavyweight broilers. These factors represent important limitations, as the findings may not be directly generalizable to extreme summer or winter weather, longer journeys, or broilers of lower slaughter weight. Even under these moderate temperatures, reducing stocking density was associated with negative effects on welfare, suggesting that longer transport distances could potentially exacerbate these adverse outcomes.

Further research is needed to confirm and extend these findings under a broader range of conditions, including different journey durations, summer and winter temperature extremes, various crate designs, and different broiler body weights. Replicating comparable large-scale studies across multiple European regions would help establish a comprehensive climatic and operational model to guide future animal welfare policy in poultry transport.

## Figures and Tables

**Table 1:** Descriptive statistics for broiler transports conducted under current EU regulation (Control truck, 160 cm^2^/kg, *N* = 7).

Variables	Mean	95% Confidence Interval for Mean		Median	Variance	Standard Error
		Lower Bound	Upper Bound			
Mean external temperature (°C)	8.60	7.06	10.14	8.00	2.79	0.63
External relative humidity (%)	75.17	71.86	78.48	77.00	12.82	1.35
Transport duration (min)	22.43	18.89	25.96	24.00	14.62	1.45
Lairage duration (min)	173.29	142.94	203.64	158.00	1076.90	12.40
Average body weight (kg/bird)	3.21	3.17	3.24	3.21	0.00	0.02
DOA birds (%)	0.36	0.32	0.40	0.36	0.00	0.02
Wing injuries (%)	4.28	3.41	5.15	3.97	0.89	0.36
Thigh injury (%)	0.98	0.44	1.51	1.08	0.34	0.22
Bruising (%)	3.40	2.64	4.17	3.25	0.69	0.31
Condemnation (%)	0.57	0.54	0.59	0.57	0.00	0.01

**Table 2:** Descriptive statistics for broiler transports conducted under EFSA-recommended density (Test truck, 200 cm^2^/kg, *N* = 7).

Variables	Mean	95% Confidence Interval for Mean		Median	Variance	Standard Error
		Lower Bound	Upper Bound			
Mean external temperature (°C)	9.49	8.35	10.62	9.70	1.50	0.46
External relative humidity (%)	73.83	71.07	76.59	73.60	8.89	1.13
Transport duration (min)	21.43	16.84	26.02	24.00	24.62	1.88
Lairage duration (min)	165.29	142.10	188.47	164.00	628.57	9.48
Average body weight (kg/bird)	3.19	3.17	3.22	3.19	0.00	0.01
DOA birds (%)	0.61	0.57	0.65	0.60	0.00	0.02
Wing injuries (%)	6.91	5.30	8.52	6.86	3.02	0.66
Thigh injury (%)	1.44	0.90	1.99	1.44	0.35	0.22
Bruising (%)	6.76	5.59	7.93	6.50	1.59	0.48
Condemnation (%)	0.78	0.70	0.86	0.81	0.01	0.03

**Table 3:** Comparative analysis between Test (200 cm^2^/kg) and Control (160 cm^2^/kg) transport groups, including effect sizes (Cohen’s d) and statistical test results.

Variable	Test Trucks (200 cm^2^/kg) (*N* = 7) Mean	Control Trucks (160 cm^2^/kg) (*N* = 7) Mean	Difference (Test– Control)	Statistical Test **	*p*-Value *	Cohen’s d (Effect Size)
Mean external temperature (°C)	9.49	8.60	0.89	Mann–Whitney U-test	0.165	0.60 (medium)
External relative humidity (%)	73.83	75.17	–1.34	Mann–Whitney U-test	0.165	–0.41 (no)
Transport duration (min)	21.43	22.43	–1.00	Mann–Whitney U-test	0.620	–0.23 (no)
Lairage duration (min)	165.29	173.29	–8.00	Mann–Whitney U-test	0.902	–0.27 (no)
Average body weight (kg/bird)	3.19	3.21	–0.01	Independent- samples *t*-test	0.539	–0.34 (no)
DOA birds (%)	0.61	0.36	0.25 *	Independent- samples *t*-test	0.000	6.17 (large)
Wing injuries (%)	6.91	4.28	2.63 *	Independent- samples *t*-test	0.004	1.88 (large)
Thigh injury (%)	1.44	0.98	0.46	Independent- samples *t*-test	0.164	0.79 (medium)
Bruising (%)	6.76	3.40	3.35 *	Independent- samples *t*-test	0.000	3.14 (large)
Condemnation (%)	0.78	0.57	0.22 *	Welch’s *t*-test	0.000	3.44 (large)

* significant difference (*p* < 0.05); ** normality and variance homogeneity were checked to select the appropriate statistical tests.

**Table 4:** Descriptive statistics for all broiler transports carried out under current EU regulation (160 cm^2^/kg, *N* = 26) used for correlation analyses.

Variables	Mean	95% Confidence Interval for Mean		Median	Variance	Standard Error
		Lower Bound	Upper Bound			
Mean external temperature (°C)	9.30	7.57	11.03	7.40	18.43	0.84
External relative humidity (%)	68.80	63.98	73.61	76.40	142.12	2.34
Transport duration (min)	24.15	23.25	25.06	25.00	5.02	0.44
Lairage duration (min)	171.65	151.37	191.93	167.50	2521.04	9.85
Average body weight (kg/bird)	3.20	3.19	3.22	3.19	0.00	0.01
DOA birds (%)	0.37	0.35	0.38	0.37	0.00	0.01
Wing injuries (%)	4.21	3.81	4.61	3.97	0.99	0.20
Thigh injury (%)	0.76	0.53	1.00	0.72	0.34	0.11
Bruising (%)	3.39	2.96	3.82	3.25	1.15	0.21
Condemnation (%)	0.54	0.52	0.56	0.55	0.00	0.01

**Table 5:** Correlations between transport conditions and animal welfare indicators in Control transports (*N* = 26).

Variables	Mean External Temperature (°C)	External Relative Humidity (%)	Transport Duration (min)	Lairage Duration (min)
**DOA birds (%)**	Spearman r = 0.155, *p* = 0.448	Spearman r = −0.095, *p* = 0.643	Spearman r = 0.032, *p* = 0.876	Pearson r = −0.199, *p* = 0.331
**Wing injuries (%)**	Pearson r = 0.166, *p* = 0.417	Pearson r = −0.110, *p* = 0.592	Pearson r = −0.023, *p* = 0.911	Pearson r = −0.176, *p* = 0.390
**Thigh injury (%)**	Spearman r = –0.252, *p* = 0.215	Spearman r = 0.425, *p* = 0.030 *	Spearman r = −0.187, *p* = 0.360	Pearson r = 0.175, *p* = 0.392
**Bruising (%)**	Pearson r = 0.040, *p* = 0.847	Pearson r = 0.017, *p* = 0.933	Pearson r = −0.069, *p* = 0.737	Pearson r = 0.096, *p* = 0.642
**Condemnation (%)**	Spearman r = 0.011, *p* = 0.958	Spearman r = 0.098, *p* = 0.632	Spearman r = 0.282, *p* = 0.163	Pearson r = 0.077, *p* = 0.710

* significant correlation (*p* < 0.05); normality of variables was assessed using the Shapiro–Wilk test. Pearson correlation was applied for normally distributed variables, and Spearman correlation was applied for non-normally distributed variables.

## Data Availability

The original contributions presented in the study are included in the article. Further inquiries can be directed to the corresponding author.
